# Risk phenotype for sarcopenia in older adults from Amazonas, Brazil; a cross-sectional study

**DOI:** 10.1371/journal.pone.0292801

**Published:** 2023-10-16

**Authors:** Alex Barreto de Lima, Ana Torres-Costoso, Vera Zymbal, Élvio Rúbio Gouveia, Fátima Baptista

**Affiliations:** 1 CIPER, Faculdade de Motricidade Humana, Universidade de Lisboa, Lisboa, Portugal; 2 Health and Social Research Center, Universidad de Castilla La Mancha, Cuenca, Spain; 3 Faculty of Physiotherapy and Nursing, Universidad de Castilla-La Mancha, Toledo, Spain; 4 ESS, Instituto Politécnico de Setúbal, Setúbal, Portugal; 5 Department of Physical Education and Sport, University of Madeira, Funchal, Portugal; 6 LARSYS, Interactive Technologies Institute, Funchal, Portugal; University of Campania Luigi Vanvitelli: Universita degli Studi della Campania Luigi Vanvitelli, ITALY

## Abstract

**Background:**

There are several markers for the suspicion, identification, and confirmation of sarcopenia.

**Objectives:**

To analyse the importance of several markers for assessing sarcopenia by classifying phenotypes based on five domains: symptomatology, muscle function, muscle mass, physical performance, and physical function.

**Methods:**

A cross-sectional study analysing 312 older adults (72.6±7.8 yrs) was conducted in Novo Aripuanã, Amazonas, Brazil. Symptoms of sarcopenia were determined with the SARC-Calf; muscle function was assessed using the 30-Chair Stand test (CST), 30-CST power, and handgrip strength (HGS) with and without normalisation for body mass/height; the skeletal muscle mass index (SMMI) was estimated from anthropometry; physical performance was determined through the 4-m gait speed (GS) and 6-min walking test (6MWT); and physical function was determined with the Composite Physical Function Scale (CPF).

**Results:**

Cluster analysis revealed two phenotypes (at risk vs not at risk for sarcopenia) and the contribution of each marker (ranged from 0 to 1). In men, the contribution of each marker was: 1 for SARC-Calf, 0.18 for SMMI, 0.09 for 30-CST power and 0.06 for HGS; in women: 1 for SARC-Calf, 0.25 for 30-CST power, 0.22 for SMMI, 0.06 for GS, 0.04 for HGS, and 0.03 for CPF. Considering the cutoff values proposed by Rikli and Jones (2013) for physical function and Cruz-Jentoft et al. (2019) for the other domains, the risk profile for sarcopenia was characterized by: high SARC-Calf in both sexes (men:51.8 vs 3.6%, p<0.001; women:71.2 vs 1.1%, p<0.001), low SMMI (men:73.2 vs 44.6%, p<0.002; women:44.1 vs 23.6%, p = 0.002); in women, low GS (38.7 vs 12.4%, p<0.001) and low CPF (29.7 vs 15.7%, p = 0.020), and no differences in HGS between groups in both sexes.

**Conclusions:**

SARC-Calf, SMMI, and 30-CST were more relevant markers for sarcopenia risk in older adults of both sexes, GS and CPF played also an important role in women.

## Introduction

Sarcopenia is a disease characterised by decreased muscle mass and muscle function [1]. Sarcopenia mainly affects older people, as ageing is a primary risk factor [2]. In turn, sarcopenia constitutes a risk for other clinical conditions, resilience to specific treatments, hospitalisations and their duration, functional dependence, and mortality, with high socioeconomic costs [3–5]. Despite the consequences, the approach to assessing muscle mass, muscle function and/or physical performance for the diagnosis of sarcopenia is still not well established [6].

The recommendation proposed by the different working groups for Sarcopenia—European (EWGSOP2 [2]), Asian (AWGS2, [7]) and American (SDOC [8]) does not is consensual regarding the assessment or not of muscle mass, EWGSOP2 and AWGS2 vs SDOC, respectively [5]. The assessment of muscle function, namely the handgrip strength (HGS) and chair raise for muscle strength, and the gait speed, SPPB, and walking distance test for physical performance, is consensual among the three groups of studies, but with differences in the form how results are expressed, precisely without or with adjustment for body dimensions (EWGSOP2 and AWGS2 vs SDOC), or differing in cutoff values (EWGSOP2 vs AWGS2 vs SDOC) [9]. For example, although the AWGS2 suggests a cutoff value of 28 kg for men and 18 kg for women for low grip strength [7], the EWGSOP2 proposes a cutoff value of 27 kg for men and 16 kg for women [2].

In addition, to objective assessments for the identification/diagnosis of sarcopenia in the older adults, a previous screening based on symptomatology/events (SARC-F) has also been proposed [10], which may or may not include a measurement of leg circumference (SARC-Calf) [11]. Considering the different screening and evaluation possibilities for sarcopenia and having the functional capacity for activities of daily living as the primary outcome, it is urgent to characterise a risk phenotype in older adults who inhabit the Amazonian tropical areas, a unique region in ethnic, sociocultural terms, economic, and macroenvironmental. This idea is supported by the multifactorial and complex nature of biological ageing [12]. Consequently, this study aimed to identify a risk phenotype for sarcopenia in older adults in Amazonas, Brazil and to analyse the relevance of risk of markers from the different domains of sarcopenia expression: symptomatology, muscle function, muscle mass, physical performance and physical function.

## Methods

### Participants

The sample consisted of 312 older adults (200 women and 112 men) living in Novo Aripuanã (Amazonas, Brazil). Participants were recruited from January 2018 to January 2020 through fliers, posters, and word-of-mouth in primary health units, public squares, churches, and other public places, and invitations were aired on local radio stations. After explaining the study procedures, all participants provided written informed consent. The evaluations were carried out on the premises of the University of the State of Amazonas, in the center of the city of Novo Aripuanã, by previously trained professionals. The study authors did not have access to information that could identify the participants individually during or after data collection. The following criteria were considered for participant’s inclusion: (1) older adults aged 60 and over, residing in the community; (2) be independent in carrying out activities of daily living; (3) moderate or high level of cognitive functioning; (4) no contraindications for physical exertion (stroke, neurological diseases, unstable chronic conditions; (5) without joint pain, chest pain, and angina pectoris [13]. The cognitive level was evaluated with the Mini-Mental State Examination (MMSE) [14]. An MMSE ≤ 15/30 points was used to exclude the participants from the study. This study was approved by the Ethics Committee of the State UEA according to the Declaration of Helsinki and Resolution 466/12 of the National Health Council, making part of the research project: "Sarcopenic Syndrome—Physical Function, Phenotype and Quality of Life in Elderly with and without Sedentary Lifestyle" (CAAE 74055517.9.0000.5016 / Referee 2.281.400).

### Symptoms of sarcopenia

To assess the symptoms of sarcopenia, the SARC-CAlf was used. The SARC-Calf combines the questions included in the SARC-F [10] adding calf circumference [11]. The SARC-CalF score ranges from 0 to 20 points, and individuals with a score ≥11 are considered to have significant symptomatology.

### Body size and composition

Anthropometric measurements were performed following the recommendations of the International Society for the Advancement of Kinanthropometry-ISAK [15] to assess body size and body composition. Body mass and height were measured using a calibrated mechanical scale (110 CH, Welmy, São Paulo, Brazil). Participants were barefoot, wearing light clothing, in an erect position, arms relaxed along the body, heels together, and occipital and gluteal regions touching the vertical ruler of the scale. The body mass index (BMI) was calculated by the ratio between body mass and body height squared (kg/m^2^). Fat mass was estimated from the equations proposed by Williams and colleagues [16] using triceps, subscapular, abdominal, and calf skinfolds.

Total muscle mass was estimated using equations with corrected arm, thigh, and calf circumferences [17]. Skeletal muscle mass index (SMMI, kg/m^2^) was used to identify reduced muscle mass according to sex and BMI as suggested by Wallowsky and colleagues: e.g., for men and women with normal BMI (18.5–24.9 kg/m^2^) an SMMI ≤ 7.6 kg/m^2^ in men and ≤5.6 kg/m^2^ in women, for overweight BMI (25–29.9 kg/m^2^) an SMMI ≤ 8.7 kg/m^2^ in men and ≤6.8 kg/m^2^ in women and for BMIs higher than 30, 35 and 40 kg/m_2_, an SMMI ≤ 9.5, 10.3 and 11.2 kg/m^2^ in men and ≤ 7.6, 8.4 and 9.2 kg/m^2^ in women, respectively [18].

### Muscle function and physical performance

The assessment of muscle function in the upper limbs and lower limbs was conducted with a handgrip test and a chair stand test, respectively. HGS (kg) was assessed twice in each hand, alternately using a dynamometer (EH10, Camry, California City, USA) with the participants seated and the arm to be assessed flexed at 90 degrees at the elbow [19,20]. The mean value of all measurements was used as the final score for each individual. Values of HGS <27kg and <16kg in men and women, respectively, indicated decreased muscle strength [2].

The chair stand test (CST) was performed for 30 seconds (30-CST) to assess lower limb muscle strength [21] and muscle power [22]. The participants performed as many repetitions as possible in this period. The test was performed in a standardised armless chair measuring 43 cm in height from a sitting position with the arm crossed over the chest. Verbal encouragement was given in assessing muscle function, and participants were allowed to try two times before the definitive measurement was recorded [22].

Physical performance, namely, gait speed and mobility, were measured using the 4 m walking speed test at the usual gait speed (4-MGS) [23] and the six-minute walk (6MWT) [13].

### Physical function

For physical function, habitual physical activity and the ability to carry out activities of daily living were evaluated. Physical activity was assessed using the validated International Physical Activity Questionnaire (IPAQ) [24]. Participants provided information about the type/intensity and duration of physical activity (walking, moderate, and vigorous physical activity)performed in the last week (if usual week) for estimation of metabolic equivalents (METs.min/week). The ability to perform basic, instrumental, and advanced activities of daily living was assessed using the Composite Physical Function (CPF) scale questionnaire, composed of 12 activities [21]. Poor functionality was identified for scores less than 14 points.

## Statistical analysis

All analyses were conducted using the SPSS software, version 26.0 (IBM Inc., Chicago, IL, USA). Values were presented descriptively, as the mean ± standard deviation (SD) for continuous variables and frequency (%) for categorical values. Unpaired t-tests and chi-square tests were used for continuous and categorical variables to compare the older adults’ characteristics according to sex. For continuous variables, analysis of variance (ANOVA) was used to test the mean differences between sarcopenia risk phenotypes (fixed factor) in terms of age, body composition, physical fitness and physical functioning (dependent variables) by sex; when statistical differences were found, the Bonferroni posthoc test was applied. For categorical variables, the chi-square test was used. Pearson’s correlation coefficient was calculated, using bivariate correlations to estimate the relationship between body composition, physical performance, muscle function, physical function, and symptomatology of sarcopenia. A two-step cluster analysis was performed by sex to rank a sarcopenia profile based on continuous variables of body composition (SMMI), physical fitness (HGS, 30-CST power and 4-MGS) and physical functioning (SARC-CalF score and CPF) of the participants. This approach for cluster analysis uses a distance measure to separate groups and then a probabilistic approach to choose the optimal subgroup model [25]. The Log-Likelihood was used as the similarity measure, and Schwarz’s Bayesian Information criterion was used to identify the automatic selection of the optimal number of clusters [26]. The silhouette coefficient, which compares the average within-cluster cohesion with the average between-cluster separation, was examined to assess the goodness of fit of the cluster solution. Values between 0.20 and 0.50 indicate a fair fit, and values of 0.50 or more indicate a good fit [27]. In addition, the identified clusters were compared on a set of diverse variables not included in the clustering algorithm to validate them as distinct subgroups.

## Results

The characterisation of the sample is described in Table 1. Men were heavier and taller than women (p <0.001) but had no BMI differences. Men had greater muscle mass, skeletal muscle mass index, and calf circumference (p <0.001). Conversely, women had a higher value in the fat mass index (p < 0.001). Regarding physical performance and muscle function, males had better scores than females on mobility (6MWT) and walking speed, on HGS and 30-CST power (regardless of normalisation) (p <0.001). In physical functioning, CPF was higher in men than women (p <0.001). Women showed a higher prevalence than men of low gait speed and significant symptomatology of sarcopenia (p <0.05).

**Table 1:** Descriptive characteristics of participants as mean ± standard deviation.

	Male (n = 112)	Female (n = 200)	p-value
Age, years	73.07±7.31	72.39±8.09	0.458
***Body Composition***			
Body Height, cm	159.99±8.26	150.10±5.67	<0.001
Body Mass, kg	69.29±11.61	60.52±12.18	<0.001
BMI, kg/m^2^	27.08±4.64	26.76±4.65	0.566
Muscle mass, kg	23.65±3.55	17.73±3.61	<0.001
SMMI, kg/m^2^	9.23±1.16	7.84±1.39	<0.001
Fat Mass Index, kg/m^2^	10.76±3.48	13.49±4.35	<0.001
Calf Circunference, cm	33.8±3.0	32.2±3.5	<0.001
***Physical Performance***			
6MWT, m	450.76±125.59	382.96±89.00	<0.001
Gait Speed, m/s	1.20±0.35	1.03±0.35	<0.001
Low Gait Speed, n (%)	16 (23.5)	52 (76.5)	0.016
***Muscle Function***			
Handgrip strength, kg	31.41±8.86	19.33±5.87	<0.001
Low Handgrip Strength, n (%)	77 (35.2)	142 (64.8)	0.677
Handgrip/body mass, kg/kg	0.45±0.11	0.33±0.10	<0.001
Handgrip/body height, kg/m^2^	19.61±5.27	12.86±3.83	<0.001
30-Chair Stand Test, n	11.08±3.34	10.74±3.16	0.365
30-CST Power (W)	43.50±18.73	31.43±13.47	<0.001
30-CST Power (W/body mass)	0.62±0.19	0.51±0.17	<0.001
30-CST Power (W/body height)	16.71±6.03	13.75±5.27	<0.001
***Physical Functioning***			
Physical Activity, MET.min/wk	2663±3897	2024±3131	0.115
CPF, pts	21.33±3.76	18.41±4.99	<0.001
***Sarcopenia Symptoms***			
SARC-CalF score, pts	6.42±5.12	7.57±5.71	0.080
SARC-CalF ≥ 11 pts, n (%)	31 (27.9)	80 (72.1)	0.029

BMI, body mass index. SMMI, skeletal muscle mass index; 6MWT, 6-minute walk test. CPF, composite physical function scale. SARC-Calf, sarcopenia screening questionnaire adding calf circumference.

Table 2 presents the bivariate correlation coefficients between body composition, physical performance, muscle function, physical function, and symptomatology of sarcopenia in men (Table 2A) and women (Table 2B). The bivariate correlations were conducted to select the most relevant variables in each domain for the cluster analysis carried out later. In both men and women, significant associations were observed between SMMI and HGS, HGS/m^2^, 30-CST Power and SARC-Calf (p<0.05). Men also showed a positive association between SMMI and habitual physical activity, and women with gait speed, 30-CST Power/m^2^ and CPF (p<0.05).

**Table 2:** **A**. Bivariate correlation coefficients between body composition, physical performance, muscle function, physical function, and symptomatology of sarcopenia–Men. **B**. Bivariate correlations coefficients between body composition, physical performance, muscle function, physical function, and symptomatology of sarcopenia–Women.

	2.	3.	4.	5.	6.	7.	8.	9.	10.	11.	12.	13.		2.	3.	4.	5.	6.	7.	8.	9.	10.	11.	12.	13.
1. BMI	0.573**	0.778**	0.081	-0.098	0.224*	0.324**	0.129	0.282**	-0.008	0.383**	-0.107	-0.275**	1. BMI	0.564**	0.912**	0.078	0.150*	0.200**	0.183**	0.127	0.536**	0.574**	0.031	0.091	-0.603**
2. SMMI	-	0.353**	-0.073	0.074	0.294**	0.364**	0.133	0.204*	0.056	0.281**	-0.010	-0.385**	2. SMMI	-	0.493**	0.048	0.179*	0.311**	0.302**	0.065	0.328**	0.338**	-0.014	0.206**	-0.477**
3. Fat Mass Index		-	0.006	-0.011	0.138	0.136	0.128	0.456**	-0.081	0.491**	-0.066	-0.361**	3. Fat Mass Index		-	0.122	0.218**	0.195**	0.159*	0.201**	0.609**	0.625**	0.023	0.103	-0626**
4. 6MWT			-	0.181	0.248**	0.204*	0.143	0.196*	-0.041	0.164	0.348**	-0.099	4. 6MWT			-	0.367**	0.262**	0.247**	0.164*	0.203**	0.196**	-0.056	0.290**	-0.257**
5. Gait Speed				-	0.357**	0.317**	0.322**	0.367**	-0.111	0.350**	0.287**	-0.123	5. Gait Speed				-	0.249**	0.218**	0.315**	0.370**	0.361**	-0.038	0.371**	-0.360**
6. HGS					-	0.973**	0.274**	0.471**	0.120	0.440**	0.188*	-0.302**	6. HGS					-	0.991**	0.159*	0.276**	0.256**	0.012	0.238**	-0.210**
7. HGS/m^2^						-	0.266**	0.359**	0.119	0.371**	0.137	-0.234*	7. HGS/m^2^						-	0.133	0.201**	0.199**	0.021	0.219**	-0.168
8. 30-CST							-	0.669**	-0.042	0.747**	0.219*	-0.186*	8. 30-CST							-	0.789**	0.837**	0.011	0.174*	-0.261**
9. 30-CST Power								-	0.031	0.972**	0.272**	-0.459**	9. 30-CST Power								-	0.984**	-0.007	0.223**	-0.554**
10. 30-CST Power/m^2^									0.047	0.047	0.247**	-0.438**	10. 30-CST Power/m^2^									-	0.007	0.218**	-0.549**
11. PA									-	-	0.055	0.093	11. PA										-	-0.049	-0.001
12. CPF											-	-0.112	12. CPF											-	-0.325**
13. SARC-CalF												-	13. SARC-CalF											-	-

BMI, body mass index; HGS, handgrip strength; 30-CST, 30-Chair Stand Test; SMMI, skeletal muscle mass index; 6MWT, 6-minute walk test. CPF, composite physical function scale. SARC-Calf, sarcopenia screening questionnaire adding calf circumference; PA, physical activity.

** p < 0.01;

*p <0.05.

**Fig 1:**
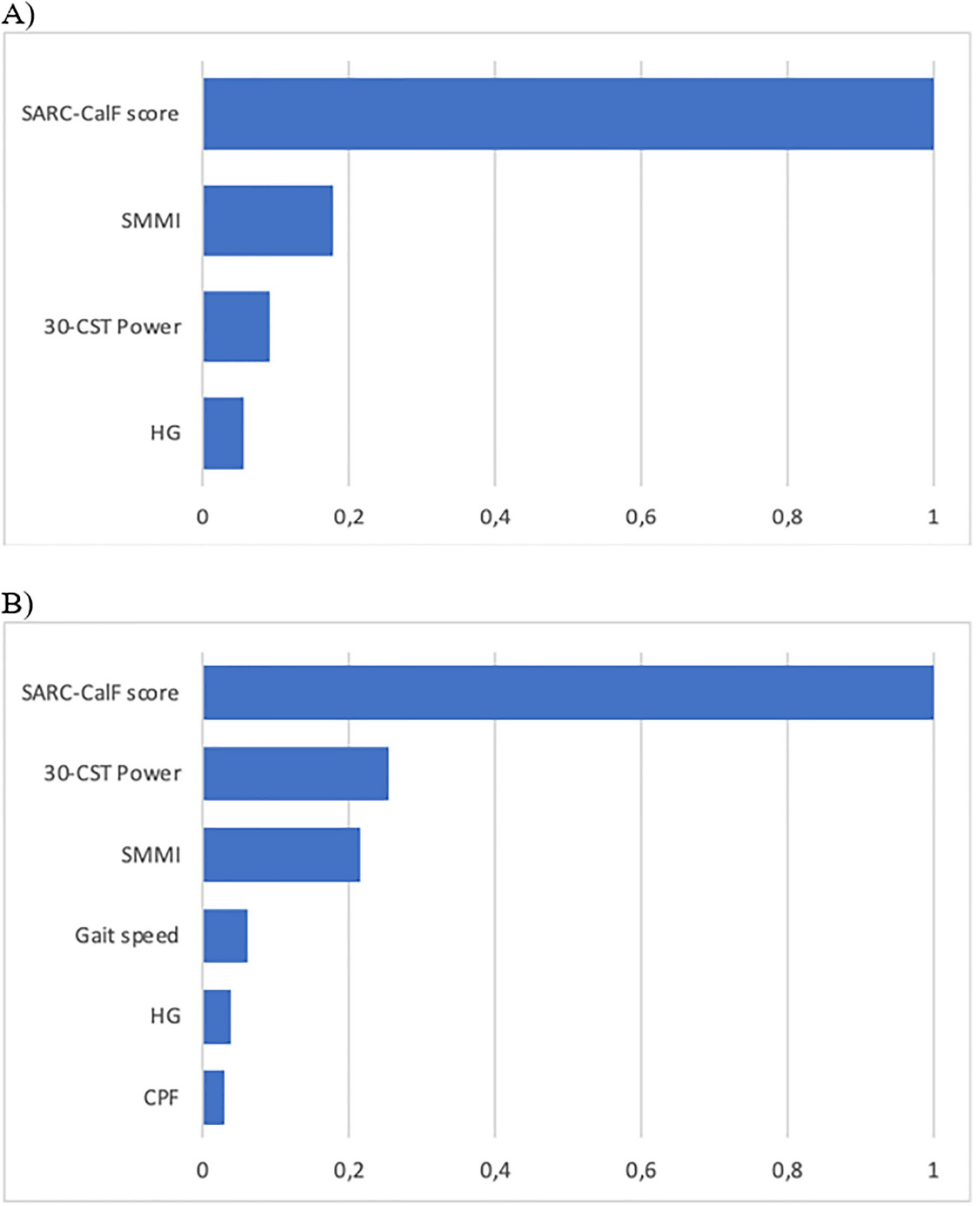
Index of the relative importance of each marker for the formation of clusters (risk vs. no risk for sarcopenia): Body composition (SMMI), muscle strength (HG, 30-CST power), physical performance (gait speed), symptomatology (SARC-CalF) and physical functioning (CPF), by sex (A: Men; B: Women). *SMMI, skeletal muscle mass index; HG, handgrip strength; 30-CST Power, 30-chair stand muscle power test; SARC-Calf, sarcopenia screening; CPF, composite physical function scale.

Fig 1 summarizes the Two-Step cluster analysis of sarcopenia markers in several domains: symptomatology (SARC-CalF), body composition (SMMI), muscle function (HG, 30-CST power), physical performance (gait speed) and physical function (CPF).

Symptomology (SARC-Calf), muscle mass (SMMI), and muscle function (30-CSt Power and HGS) were the most relevant markers for the configuration of risk clusters for sarcopenia (risk vs no risk) in both men and women and physical performance (gait speed) and physical function (CPF) in women.

Table 3 compares body composition, physical fitness, physical functioning and symptomatology of sarcopenia between two clusters to validateits identification in men (panel A) and women (panel B): cluster 1 representing a phenotype without risk for sarcopenia and cluster 2 representing a risk phenotype for sarcopenia. Differences were observed between the groups in the previously evidenced variables for the formation of clusters, with more unfavourable results for cluster 2 in both sexes. The prevalence of low SMMI and high SARC-Calf was higher in the risk cluster in both genders (men: 73.2 vs 44.6%, p = 0.002; women: 44.1 vs 23.6%, p = 0.002) and in women, it was even observed a higher prevalence of low gait speed in the risk cluster compared to the non-risk cluster (38.7 vs 12.4%).

**Table 3:** **A**. Comparison of body composition, physical fitness and physical functioning between phenotypes according to risk for sarcopenia–Men. **B**. Comparison of body composition, physical fitness and physical functioning between phenotypes according to risk for sarcopenia–Women.

Men	Cluster 1 W/O Sarcopenia Risk (n = 56)	Cluster 2 Sarcopenia Risk (n = 56)	p-value	Women	Cluster 1 W/O Sarcopenia Risk (n = 89)	Cluster 2 With Sarcopenia Risk (n = 111)	p-value
Age, years	71.41±6.55	74.73±7.71	0.016	Age, years	70.21±6.73	74.13±8.67	0.001
***Body Composition***				***Body Composition***			
Body Mass, kg	75.59±10.87	63.00±8.56	<0.001	Body Mass, kg	70.04±10.07	52.97±7.51	<0.001
Body Height, cm	161.80±9.38	158.19±6.58	0.020	Body Height, cm	152.63±5.46	148.07±5.02	<0.001
BMI, kg/m^2^	29.01±5.03	25.16±3.28	<0.001	BMI, kg/m^2^	30.06±3.99	24.12±3.27	<0.001
Muscle mass, kg	25.67±3.18	21.62±2.64	<0.001	Muscle mass, kg	20.26±3.71	15.70±1.81	<0.001
SMMI, kg/m^2^	9.82±1.09	8.63±0.92	<0.001	SMMI, kg/m^2^	8.69±1.54	7.16±0.76	<0.001
Low SMMI, n (%)	25 (44.6)	41 (73.2)	0.002	Low SMMI, n (%)	21 (23.6)	49 (44.1)	0.002
Fat Mass Index, kg/m^2^	12.25±3.48	9.26±2.79	<0.001	Fat Mass Index, kg/m^2^	16.71±3.67	10.92±2.90	<0.001
Calf circunference, cm	36.05±2.16	31.67±1.93	<0.001	Calf Circunference, cm	35.22±2.52	29.81±2.06	<0.001
***Physical Performance***				***Physical Fitness***			
6MWT, m	456.52±128.54	455.00±123.45	0.630	30-Chair Stand Test, n	11.66±3.18	9.99±2.95	<0.001
Gait Speed, m/s	1.22±0.33	1.17±0.38	0.568	6MWT, m	399.27±90.65	369.87±85.83	0.020
Low Gait Speed, n (%)	6 (10.7)	11 (19.6)	0.188	Gait Speed, m/s	1.14±0.28	0.93±0.37	<0.001
***Muscle Function***				Low Gait Speed, n (%)	11 (12.4)	43 (38.7)	<0.001
Handgrip strength, kg	33.84±9.69	28.98±7.24	0.003	Handgrip strength, kg	20.81±6.26	18.14±5.27	0.001
Low Handgrip Strength, n (%)	43 (76.8)	34 (60.7)	0.067	Low Handgrip Strength, n (%)	69 (77.5)	73 (65.8)	0.068
Handgrip/body mass, kg/kg	0.45±0.11	0.47±0.12	0.404	Handgrip/body mass, kg/kg	0.30±0.11	0.34±0.09	0.004
Handgrip/body height, kg/m^2^	20.90±5.65	18.32±4.56	<0.009	Handgrip/body height, kg/m^2^	13.62±4.04	12.26±3.55	0.011
30-Chair Stand Test, n	11.36±3.52	10.80±3.17	0.384	30-CST Power, W	40.23±13.46	24.36±8.38	<0.001
30-CST Power, W	50.31±21.93	36.67±11.48	<0.001	30-CST Power/body mass, W/kg	0.57±0.17	0.46±0.14	<0.001
30-CST Power/body mass, W/kg	0.65±0.21	0.58±0.16	0.050	30-CST Power/body height, W/m^2^	17.12±5.08	11.04±3.60	<0.001
30-CST Power/body height, W/m^2^	18.85±6.87	14.56±4.10	<0.001				
***Physical Functioning***				***Physical Functioning***			
Physical Activity, MET.min/wk	2479±3371	2890±4390	0.582	Physical Activity, MET.min/wk	1924±3054	2103±3204	0.690
CPF, pts	21.00±4.20	21.66±3.26	0.355	CPF, pts	19.49±4.74	17.54±5.04	0.006
CPF score, n (%)	6 (10.7)	2 (3.6)	0.142	CPF score, n (%)	14 (15.7)	33 (29.7)	0.020
***Sarcopenia Symptoms***				***Sarcopenia Symptoms***			
SARC-CalF score, pts	1.92±2.62	10.92±2.21	<0.001	SARC-CalF score, pts	1.89±2.42	12.12±2.70	<0.001
SARC-CalF ≥ 11, n (%)	2 (3.6)	29 (51.8)	<0.001	SARC-CalF ≥ 11 pts, n (%)	1 (1.1)	79 (71.2)	<0.001

BMI, body mass index. SMMI, skeletal muscle mass index; 6MWT, 6-minute walk test. CPF, composite physical function scale. SARC-Calf, sarcopenia screening questionnaire adding calf circumference.

## Discussion

Considering the various evaluation possibilities, from screening and identification to confirmation and severity of sarcopenia, this investigation aimed to identify a risk phenotype in a sample of older adults in Amazonas, Brazil. The results suggest symptomatology (SARC-Calf), muscle mass (SMMI) and muscle function (30-CST Power, HGS) as the most relevant markers for a sarcopenia risk phenotype in men and women and, additionally, physical performance (speed gait) and physical function (ability to perform activities of daily living) for the risk of sarcopenia in women.

The grouping of these variables to classify people based on the observation of similarities and dissimilarities concerning sarcopenia thus proved to be different for men and women. Gait slowness was more prevalent in women than men, while muscle weakness was more prevalent in men in our sample [28]. These observations may be due to a lower gait speed in women and a more significant age-related decline in muscle mass/strength in men [29]. The present study reinforces the relevance of gait speed and the ability to perform activities of daily living in conjunction with symptomatology, muscle mass, and muscle strength to identify women at risk for sarcopenia.

Compared to elderly men, elderly women generally have a lower gait speed [30–32]. The difference between sexes is clinically relevant since it equals the minimal clinically significant individual differences estimated for gait speed, ranging between 0.03 and 0.05 m/s [31]. In this sense, for the same cutoff value, the prevalence of slow gait may likely be higher in women than in men. However, in women than men, gait speed seems more affected by physical inactivity [33] and overweight/obesity [34].

Although several behavioral, hormonal, and structural factors have been suggested to explain differences in walking speed between elderly women and men elderly, it is necessary to go further on this topic [34,35]. Women also show lower levels of physical functioning than men [36] and a faster or possibly earlier decline due to menopause [37].

Getting up from a sitting position is a prerequisite for walking and performing activities of daily living, which require more muscle power than muscle strength [2]. The 30s-CST is a simple test widely used in clinical and laboratory settings [38,39] and can be used to estimate lower limb power [40–42].

The role of muscle strength vs muscle power in sarcopenia has recently been discussed. Lower limb muscle power decreases earlier and faster when compared to muscle strength and seems more strongly associated with disability, hospitalisation and mortality [42,43]. Lower limb muscle power also appears to respond better to resistance exercise interventions in older adults at risk of sarcopenia than handgrip strength [41]. Our findings corroborate the literature showing the importance of evaluating the muscle power of the lower limbs in older adults [44]. However, this evaluation can take on different formats, including vertical jump power in older adults who can perform this test safely [45].

According to the objective of the work (identification of risk phenotypes), the main limitations concern the methodology for evaluating some variables, namely muscle mass and muscle power of the lower limbs, both estimated and not adequately evaluated. The total SMMI was determined from anthropometric measurements [17] and the identification of low SMMI using cutoff values that were established using another method (bioimpedance) and population (Caucasian) [18]. The selection of this approach to identify low SMMI was because the magnetic resonance of the whole body validated the equations for estimating SMMI through anthropometric measurements or by bioimpedance [46]. The muscle power of the lower limbs was also estimated from an equation developed for the 30-CST validated by assessments conducted in instrumented leg press and different force platforms [44,47], but in samples that may have characteristics different from ours. Another limitation is the cross-sectional design of the investigation, which does not allow for inducing causality. Despite the limitations, this investigation established a risk profile identifying the importance of several sarcopenia markers. More studies are needed to clarify the best approach to anticipate sarcopenia in this population.

## Conclusions

In conclusion, the present study showed that SARC-Calf (symptomology), SMMI (muscle mass), and lower limb power (muscle function) might be the primary risk markers for sarcopenia in both men and women in Amazonas, Brazil, with muscle power in the lower limbs supplanting the relevance of muscle mass to the risk of sarcopenia in women but not in men.

## Supporting information

S1 Data (XLSX)
